# The validity and reliability of the Swedish CancerSupportSource-Caregiver: a screening tool for psychological distress and support needs in clinical cancer care

**DOI:** 10.1007/s00432-026-06495-9

**Published:** 2026-05-13

**Authors:** Maria Samuelsson, Marie-Louise Möllerberg, Benjamin Maus, Carl Magnus Olsson, Jenny Jakobsson

**Affiliations:** 1 Department of Care Science, Faculty of Health and Society, Malmö University, Jan Waldenströms gata 25, 205 06 Malmö, Sweden; 2 Department of Computer Science and Media Technology, Faculty of Technology and Society, Malmö University, Bassänggatan 2, 211 19 Malmö, Sweden; 3 CaRe – Cancer and Illness Rehabilitation Research Group, Malmö University, Jan Waldenströms gata 25, 205 06 Malmö, Sweden; 4 Sustainable Digitalization Research Centre, Malmö University, Bassänggatan 2, 211 19 Malmö, Sweden

**Keywords:** Cancer, Caregiver, Distress, Psychometrics, Screening, Support

## Abstract

**Background:**

Timely, individually tailored support for family caregivers of cancer patients is stressed, reinforcing the importance of implementing screening tools in clinical practice.

**Aim:**

This study aims to evaluate the validity and reliability of the Swedish CancerSupportSource-Caregiver among 145 Swedish family caregivers of persons diagnosed with cancer.

**Methods:**

We evaluated the validity and reliability of the Swedish CancerSupportSource-Caregiver among 145 Swedish family caregivers of persons diagnosed with cancer who responded to the Swedish CancerSupportSource-Caregiver, sociodemographic questions, and the Hospital Anxiety and Depression Scale. Psychometric analyses were performed using descriptive statistics and classical test theory to evaluate data quality, targeting, scaling assumptions, and internal validity. Construct validity was assessed through confirmatory factor analysis; criterion validity through concurrent validity; and reliability through internal consistency.

**Result:**

Overall, in the sample, evaluations demonstrated generally satisfactory psychometric properties with respect to data quality, targeting, and scaling assumptions. The hypothesized five-domain model showed an acceptable fit to the data, although there were indices that it could be improved. Item loadings were generally high, supporting the proposed construct structure. Further, assessments of the criterion validity were satisfactory. However, the evaluations of internal validity and internal consistency indicated redundancy, mainly within the *emotional well-being* domain.

**Conclusion:**

The Swedish CancerSupportSource-Caregiver demonstrated preliminary satisfactory abilities to screen for support needs and psychological distress among Swedish family caregivers of persons diagnosed with cancer. Further evaluations in larger samples, using Rasch measurement theory, could provide a deeper understanding of the functioning of items and response options.

## Introduction

Globally, the incidence and prevalence of cancer are expected to rise in the coming decades (World Health Organisation 2021), leading to an increase in co-affected family caregivers (Coyne et al. 2020). Family caregivers of persons diagnosed with cancer face a higher risk of negative health outcomes, such as psychological distress and stress-related illness, compared to the general population (Mollerberg et al. 2016; Hu et al. 2023). These negative health outcomes may lead to individual and family suffering (Samuelsson et al. 2024) and broader societal costs (Socialstyrelsen 2021). For instance, illness among family caregivers may result in increased health care use, sick leave from work, and decreased caregiving capacity. Importantly, family caregiving plays a critical role in cancer patient survival (Krajc et al. 2023) and quality of cancer care (Samuelsson et al. 2022). For instance, family caregivers monitor side effects and complications of treatments, support treatment compliance, and support the patient's rehabilitation after treatment. All of which underscores the need to support family caregivers throughout the cancer trajectory.

However, family caregivers’ support needs are complex and influenced by multiple factors, including not only the patient’s prognosis and treatment but also the family caregiver’s own characteristics, social context, and available resources (Krishnasamy et al. 2022). Accordingly, family caregivers of persons diagnosed with cancer require relevant and timely support tailored to individual needs (Howard et al. 2022; Lambert et al. 2019). For this purpose, screening tools for clinical use are essential. Support needs screening differs from comprehensive needs assessment in that it aims to briefly and efficiently identify family caregivers at risk of unmet needs or psychological distress, thereby enabling timely guidance or referral rather than providing an in-depth exploration of needs. As of today, there is a lack of Swedish-language screening instruments designed for routine cancer care.

The CancerSupportSource-Caregiver (CSS-C) is a validated, multidimensional digital screening tool designed to assess family caregivers’ support needs and psychological distress and provide automated triaging and tailored referrals to support services (Zaleta et al. 2023). The CSS-C exists in two versions, one with 33 items (Applebaum et al. 2024) and a more recent version with 19 items (Zaleta et al. 2023). This study is based on the 19-item version. The original CSS-C comprises five domains: *patient well‐being*, *healthy lifestyle*, *caregiving tasks*, *emotional well‐being*, and *finances*. The *emotional well-being* domain comprises two subscales (anxiety and depression), each with two items assessing psychological aspects (two for anxiety and two for depression). Furthermore, CSS-C includes an item that assesses concerns related to the use of tobacco, alcohol, or other substances, not included in any of the five domains. The original CSS-C has demonstrated satisfactory psychometric properties, feasibility, and acceptability across several contexts, including instrument development and psychometric validation in community-based samples, assessments of feasibility and acceptability in routine cancer care, and use as part of structured distress screening and referral interventions in specialized cancer settings (Shaffer et al. 2019; Zaleta et al. 2023; Applebaum et al. 2024). Evaluations of the original CSS-C have shown high internal consistency (Cronbach’s alpha = 0.92), a test‐retest reliability of 0.85, and confirmed multi‐dimensionality (inter‐factor correlations ranging from 0.26 to 0.61). Moreover, the total distress score of the anxiety and depression subscales strongly correlated with each domain (*r* = 0.52–0.83; *p* < 0.001) (Zaleta et al. 2023).

We undertook a thorough translation and cultural adaptation of the CSS-C from English to Swedish (submitted). In our translation and adaptation study, two expert panels comprising healthcare professionals and family caregivers evaluated the content validity of the translated version of the CSS-C through cognitive interviews and content validity index assessments. Based on their feedback, empirical evidence, and considerations of the Swedish tax-financed healthcare system, one item from the original CSS-C (“Managing health insurance and medical bills”) was excluded in the Swedish version, and six items were added: “Getting enough sleep”, “Your own health”, “Managing the household”, “Other family member’ well-being”, and “Feelings of fatigue”. Consequently, the Swedish CSS-C comprises 24 items. Table 2 demonstrates the items and domains of the Swedish version. In addition, the original domain Finances, which consisted of two items (“Managing household finances” and the excluded item “Managing health insurance and medical bills”), was reconceptualized into a broader Family Life domain, incorporating both retained and newly added items. This revision was aimed at better capturing family‑related concerns considered relevant in the Swedish context. The descriptive pilot evaluation (data quality, scaling assumption, targeting, criterion validity, and internal validity) of the translation and cultural adaptation indicated promise for identifying support needs and symptoms of anxiety and depression among Swedish family caregivers of persons diagnosed with cancer. Further, healthcare professionals assessed the Swedish version as relevant and useful. However, the pilot evaluation did not permit further psychometric testing. Hence, formal assessments of the validity and reliability of the Swedish CSS-C and its domains are needed.

The Swedish CSS-C is intended for routine cancer care and will be offered to the patient’s family caregivers by the patient’s cancer nurse. Family caregivers can use the Swedish CSS-C to self-assess their support needs and receive tailored support recommendations (e.g., a conversation with the healthcare professionals caring for the patient, contact with a healthcare center to address concerns about their own health, or access to tailored evidence-based informational resources). The intended function of the Swedish CSS-C is as a digital self-directed screening and self-care support tool rather than a clinical assessment instrument. Given that Swedish healthcare services have no legal responsibility for patients’ family caregivers, healthcare professionals do not have access to family caregivers’ CSS-C responses. The digital version has been co-designed together with family caregivers, and its functioning and usability have been tested, showing promising results (submitted). Accordingly, the purpose of the Swedish CSS-C is to prevent negative health outcomes by enabling family caregivers to identify their own support needs and facilitating access to relevant resources and support.

## Objective

This study aims to evaluate the validity and reliability of the Swedish CSS-C as a screening tool for psychological distress and support needs among Swedish family caregivers of persons diagnosed with cancer.

## Materials and methods

### Design

This study was designed as a psychometric evaluation study using questionnaire data from 145 family caregivers to evaluate the validity and reliability of the Swedish CSS-C through classical test theory**.**

### Data collection

To evaluate whether the Swedish CSS-C can identify family caregivers’ support needs, we collected data from a sample of Swedish family caregivers of persons diagnosed with cancer, who responded to the Swedish CSS-C and sociodemographic questions. Criterion validity with respect to psychological distress was assessed using concurrent validity; therefore, the participants also completed the Hospital Anxiety and Depression Scale (HADS) (Zigmond and Snaith 1983). Family caregivers were recruited through posters in the waiting rooms of five oncological/surgical clinics in southern Sweden from November 2024 to November 2025. In addition, digital posters were used in a Swedish online forum for family caregivers of persons diagnosed with cancer. All posters included brief information on the study and a QR code that linked to a university-provided survey tool; further information on the study’s aims, procedures, voluntariness, and contact information for the research team; and a consent form. Those interested could, after providing informed consent, complete the questionnaires. For sample size, we followed the recommendations in the COSMIN checklist that a sample of at least 100 participants is sufficient to yield methodologically sound estimates (Gagnier et al. 2021).

### Participants

The inclusion criteria for participation in the psychometric evaluation of the Swedish CSS-C were being a family caregiver of a person diagnosed with cancer, being at least 18 years old, and being able to read and understand Swedish. In this study, “family caregiver” was defined broadly to include individuals regardless of biological, legal, or social relationship to the patient, in accordance with a family-centered perspective (Shajan and Snell 2019).

### Analysis

Psychometric analyses of the Swedish CSS-C were performed in IBM SPSS Statistics 28, using descriptive statistics and classical test theory. Construct validity was assessed through confirmatory factor analysis (CFA) using IBM SPSS AMOS 30. Criterion validity was evaluated using concurrent validity analyses, and reliability was assessed through measures of internal consistency (Hobart et al. 2004; Streiner and Norman 2014). In accordance with recommendations from the developers of the original CSS-C (Zaleta et al. 2023), Item 24 (“Tobacco, alcohol, or other substance use”) was not included in any of the instrument’s domains and was only evaluated using descriptive statistics.

#### Data quality

To evaluate data quality, we determined the proportion of missing data per item and the percentage of computable scale scores (Hobart et al. 2004).

#### Targeting

To evaluate whether the Swedish CSS-C could target the full variance within the sample, we calculated ceiling and floor effects and skewness. Ceiling and floor effects were considered present if the proportion of response alternatives per item was > 90% and the skewness was outside the range − 1 to 1 (Ware and Gandek 1998; Hobart et al. 2004).

#### Scaling assumptions

To evaluate whether items could be summed within their allocated domains, we reviewed item means, standard deviations, and response distributions within each domain and calculated item-total correlations. In Likert scales, item distributions and variability should be broadly comparable within domains (Hobart et al. 2004). Further, all items should contribute equally and substantially (*r* ≥ 0.30) to the total score of their domain.

#### Internal validity

To evaluate internal validity, we determined whether the domains measured distinct yet similar aspects of the underlying construct. Thus, following Hobart et al. (2004), we determined intercorrelations using Pearson’s correlation coefficient, expecting moderate correlations (*r* = 0.30–0.70).

#### Criterion validity

To evaluate the criterion validity of the subscales intended to indicate anxiety and depression, we used concurrent validity, with the HADS as a gold standard. Accordingly, we used Spearman’s rank correlation coefficients to examine associations between the total scores of the Swedish CSS-C anxiety and depression subscales included in the *emotional well-being* domain and the corresponding HADS subscale scores. In accordance with Swinscow and Campbell (1997), a priori hypotheses specified that correlations of ≥ 0.40 would indicate satisfactory criterion validity, with statistical significance defined at *p* < 0.05.

The HADS is a self-report instrument consisting of 14 items designed to assess the presence and severity of anxiety and depression symptoms (Zigmond and Snaith 1983). It includes two subscales, each with seven items rated on a four-point Likert scale (0 = *not at all* to 3 = *very much*). Each subscale yields a score ranging from 0 to 21, with higher scores indicating more symptoms of anxiety or depression. Both subscales exhibited a high degree of internal consistency, with Cronbach’s alpha values ranging from 0.71 to 0.90. The HADS has been validated across various populations, including cancer patients (Bjelland et al. 2002) and their family caregivers (Zigmond and Snaith 1983). Cronbach’s alpha values among family caregivers of cancer patients represent 0.85 for the anxiety subscale and 0.84 for the depression subscale (Gough and Hudson 2009).

#### Construct validity

To confirm the hypothesized five-domain model (*patient well-being*, *healthy lifestyle*, *caregiving tasks*, *family life*, and *emotional well-being*) of the Swedish CSS-C, we conducted a CFA using maximum-likelihood estimation following the cut-off criteria for fit indices by Hu and Bentler (1999) and Browne and Cudeck (1993). Missing data were handled using Full Information Maximum Likelihood (FIML) estimation in AMOS. We evaluated how well the hypothesized model fitted the data through relative/normed chi-square statistics (CMIN/DF), goodness-of-fit statistics (GFI), the root mean square error of approximation (RMSEA), and the standardized root mean square residual (SRMR). To test the hypothesis that all domains are uncorrelated, we analyzed the comparative fit index (CFI). Furthermore, item loadings were examined and expected to be high (> 0.70) in their allocated domain.

#### Convergent validity

To evaluate the convergent validity, we calculated the average variance extracted (AVE) per domain, with the criterion AVE > 0.50 indicating that a domain explains more variance in its items than is attributable to measurement error (Fornell and Larcker 1981; Hair et al. 2019).

#### Discriminant validity

To evaluate discriminant validity, we compared the square root of each domain’s AVE with its correlations to all other domains. Following the Fornell and Larcker criteria (1981), discriminant validity was affirmed when the square root of AVE for a specific domain surpassed all of its inter-domain correlations.

#### Reliability

To evaluate the internal consistency of the Swedish CSS-C and its domains, we calculated corrected item-total correlations, Cronbach’s alpha, and homogeneity. Following Hobart et al. (Hobart et al. 2004), the cut-off criteria were > 0.40 for satisfactory item-total correlations, 0.70 for Cronbach’s alpha, and > 0.30 for homogeneity (Ware and Gandek 1998).

## Results

In total, 145 family caregivers of persons diagnosed with cancer responded to the Swedish CSS-C, sociodemographic data, and HADS. The participants’ characteristics are shown in Table 1.

**Table 1:** The sociodemographic characteristics of the 145 participants

	Number	%
*Sex*		
Female	123	84.8
Male	21	14.5
Other	0	
Missing	1	
*Age in years*		
18–30	8	5.5
31–40	15	10.3
41–50	35	24.1
51–60	49	33.8
61–70	30	20.7
71–80	8	5.5
81 and above	0	
*Education*		
Nine-year compulsory school	8	5.5
Upper secondary school	39	26.9
Higher education	97	66.9
Missing	1	
*Relation*		
Partner	91	62.8
Child	31	21.4
Sibling	5	3.4
Parent	13	9.0
Relative	2	1.4
Friend	3	2.1

### Data quality

The proportion of missing data per item was low (0.7%) and distributed across items (Table 2). All items were endorsed by 97% of the participants. The computable scale scores were 100%. This indicates high data quality.

**Table 2:** Evaluations of data quality and targeting of the Swedish CSS-C among n = 145 Swedish family caregivers of persons diagnosed with cancer

Item	Item frequency distribution in %						Item descriptive statistics		
	Missing data n (%)	Not at all	Slightly	Moderate	Seriously	Very seriously	Mean	SD	Skewness
*First, we want to understand your concerns about THE PATIENT. Today, how CONCERNED are you about…*									
(1) The patient’s eating and nutrition	0	27.6	19.3	20.0	20.0	13.1	2.72	1.398	0.193
(2) Changes in the patient’s mood or behavior	1 (0.7)	11.0	22.1	31.0	23.4	11.7	3.03	1.176	− 0.028
(3) Changes in the patient’s memory or thinking	0	24.8	24.1	25.5	15.9	9.7	2.61	1.281	0.315
(4) The patient’s pain or physical discomfort	1 (0.7)	7.6	18.6	23.4	24.8	24.8	3.41	1.259	− 0.283
(5) Your relationship^†^	1 (0.7)	35.2	23.4	13.8	15.2	11.7	2.44	1.408	0.542
*Next, we want to understand your concerns about YOURSELF. Today, how CONCERNED are you about…*									
(6) Eating and nutrition	0	49.7	24.8	12.4	10.3	2.8	1.92	1.133	1.064
(7) Exercising and being physically active	0	28.3	26.2	18.6	20.7	6.2	2.50	1.270	0.343
(8) Getting enough sleep^†^	1 (0.7)	17.9	24.8	24.8	20.0	11.7	2.83	1.276	0.147
(9) Your own health^†^	0	13.8	27.6	33.8	15.2	9.7	2.79	1.154	0.249
(10) Keeping up with your health care needs	0	24.1	26.2	29.0	13.1	7.6	2.54	1.208	0.378
(11) Providing transportation to treatment and appointments	0	36.6	23.4	21.4	11.7	6.9	2.29	1.263	0.631
(12) Making treatment decisions	0	22.1	29.7	27.6	12.4	8.3	2.55	1.201	0.437
(13) Coordinating medical care for the patient	1 (0.7)	20.7	30.3	22.1	13.8	12.4	2.67	1.296	0.409
(14) Providing physical or medical care to the patient	0	31.0	22.8	24.1	15.2	6.9	2.44	1.263	0.417
(15) Managing the household^†^	0	26.2	29.7	20.0	13.8	10.3	2.52	1.297	0.498
(16) Managing household finances	0	23.4	20.0	26.2	15.2	15.2	2.79	1.365	0.195
(17) Other family members’ well-being^†^	0	4.8	21.4	32.4	21.4	20.0	3.30	1.157	− 0.016
(18) Changes or disruptions in work, school, or home life	0	9.7	17.9	30.3	26.2	15.9	3.21	1.195	− 0.186
(19) Feeling sad or depressed^††^	0	6.2	13.8	26.2	31.0	22.8	3.50	1.167	− 0.433
(20) Feeling nervous or afraid^†††^	0	2.1	11.7	26.2	27.6	32.4	3.77	1.093	− 0.459
(21) Worrying about the future and what lies ahead^†††^	1 (0.7)	1.4	4.8	20.0	26.2	46.9	4.13	0.991	− 0.924
(22) Feeling lonely or isolated^††^	0	13.1	21.4	17.9	22.8	24.8	3.25	1.382	− 0.184
(23) Feelings of fatigue^†^	1 (0.7)	9.0	18.6	29.0	21.4	21.4	3.28	1.248	− 0.149
(24) Tobacco, alcohol, or other substance use	0	71.7	17.2	6.2	2.8	2.1	1.46	0.890	2.244

^†^Added to the Swedish version based on feedback from expert panels of healthcare professionals and family caregivers. ^††^Depression subscale ^†††^Anxiety subscale

### Targeting

Descriptive statistics showed no ceiling or floor effects (Table 2). The frequencies for item-response options were well distributed across categories. Overall, the item-response frequency distribution was symmetrical, except for Items 6 (“Eating and nutrition”) and 24 (“Tobacco, alcohol, or other substance use”), which were right-skewed. In addition, the skewness of Item 21 (“Worrying about the future and what lies ahead”) was − 0.924. Scale skewness was 0.124. These findings indicate overall successful targeting.

### Scaling assumptions

Item mean scores for the total scale ranged from 1.46 to 4.13, with Item 24 (“Tobacco, alcohol, or other substance use”) showing the lowest mean (1.46) and Item 21 (“Worrying about the future and what lies ahead”) showing the highest mean (4.13) (Table 2). Responses were distributed across all response categories. Item mean scores and standard deviations across domains were relatively equivalent (Table 3), and all items demonstrated corrected item–total correlations exceeding 0.30, indicating that each item contributed meaningfully to its allocated domain. These findings support the summation of items within domains.

**Table 3:** Evaluations of scaling assumptions, internal validity, and internal consistency of the Swedish CSS-C among n = 145 Swedish family caregivers of persons diagnosed with cancer

Domains	Mean	SD	Item total correlations	Scale mean if item deleted	Scale variance if item deleted	Cronbach’s alpha if item deleted
*Patient well-being*						
The patient’s eating and nutrition	2.71	1.399	0.561	9.04	8.488	0.685
Changes in the patient’s mood or behavior	3.03	1.176	0.581	8.72	9.433	0.676
Changes in the patient’s memory or thinking	2.60	1.280	0.489	9.15	9.510	0.723
The patient’s pain or physical discomfort	3.41	1.259	0.559	8.34	9.163	0.685
	Cronbach’s alpha 0.75 Homogenity 0.43					
*Healthy lifestyle*						
Eating and nutrition	1.92	1.135	0.688	10.67	16.781	0.853
Exercising and being physically active	2.51	1.268	0.684	10.08	15.937	0.855
Sleep^†^	2.83	1.276	0.651	9.77	16.164	0.863
Own health^†^	2.79	1.158	0.789	9.81	15.850	0.829
Keeping up with your health care needs	2.54	1.211	0.723	10.06	15.997	0.845
	Cronbach’s alpha 0.88 Homogenity 0.59					
*Caregiving tasks*						
Providing transportation to treatment and appointments	2.30	1.263	0.626	7.66	10.394	0.817
Making treatment decisions	2.55	1.205	0.652	7.41	10.537	0.805
Coordinating medical care for the patient	2.67	1.296	0.665	7.29	9.970	0.800
Providing physical or medical care to the patient	2.44	1.267	0.748	7.51	9.608	0.762
	Cronbach’s alpha 0.84 Homogenity 0.57					
*Family life*^††††^						
Your relationship^†^	2.44	1.408	0.401	11.85	14.769	0.765
Managing the household^†^	2.53	1.301	0.516	11.77	14.318	0.720
Managing household finances	2.80	1.362	0.613	11.50	13.175	0.683
Other family members´s well-being^†^	3.32	1.144	0.468	10.98	15.559	0.736
Changes or disruptions in work, school, or home life	3.21	1.200	0.666	11.09	13.733	0.669
	Cronbach’s alpha 0.76 Homogenity 0.39					
*Emotional well-being*						
Feeling sad or depressed^††^	3.51	1.174	0.878	14.44	17.248	0.895
Feeling nervous or afraid^†††^	3.77	1.099	0.866	14.18	17.924	0.898
Worrying about the future and what lies ahead^†††^	4.13	0.995	0.744	13.82	19.643	0.922
Feeling lonely or isolated ^††^	3.25	1.386	0.813	14.70	16.198	0.911
Feelings of fatigue^†^	3.29	1.248	0.766	14.66	17.605	0.917
	Cronbach’s alpha 0.93 Homogenity 0.72					

^†^Added to the Swedish version based on feedback from expert panels of healthcare professionals and family caregivers. ^††^Depression subscale ^†††^Anxiety subscale ^††††^New domain in the Swedish version

### Internal validity

Internal validity was supported by corrected item–total correlations exceeding 0.30 across all items (Table 3). However, eight items exceeded 0.70, indicating potential redundancy. All items within the *emotional well-being* domain exceeded this threshold, suggesting substantial overlap among items within this domain.

### Criterion validity

Associations between the Swedish CSS-C anxiety and depression subscales and the corresponding HADS subscales were strong and positive (Table 4). In addition, notable cross-domain correlations were observed between the Swedish CSS-C depression scale and the HADS anxiety scale (r = 0.71), as well as between the Swedish CSS-C anxiety scale and the HADS depression scale (r = 0.68).

**Table 4:** Correlations between the anxiety and depression subscales of the Swedish CSS-C and the HADS

	The Swedish CSS-C anxiety subscale	The Swedish CSS-C depression subscale
HADS Anxiety subscale	0.720*	–
HADS Depression subscale	–	0.757*

HADS = hospital anxiety and depression scale. CSS-C = Cancer Support Source-Caregivers

*Correlation is significant, with a *p* value of 0.01

### Construct validity

**Fig. 1:**
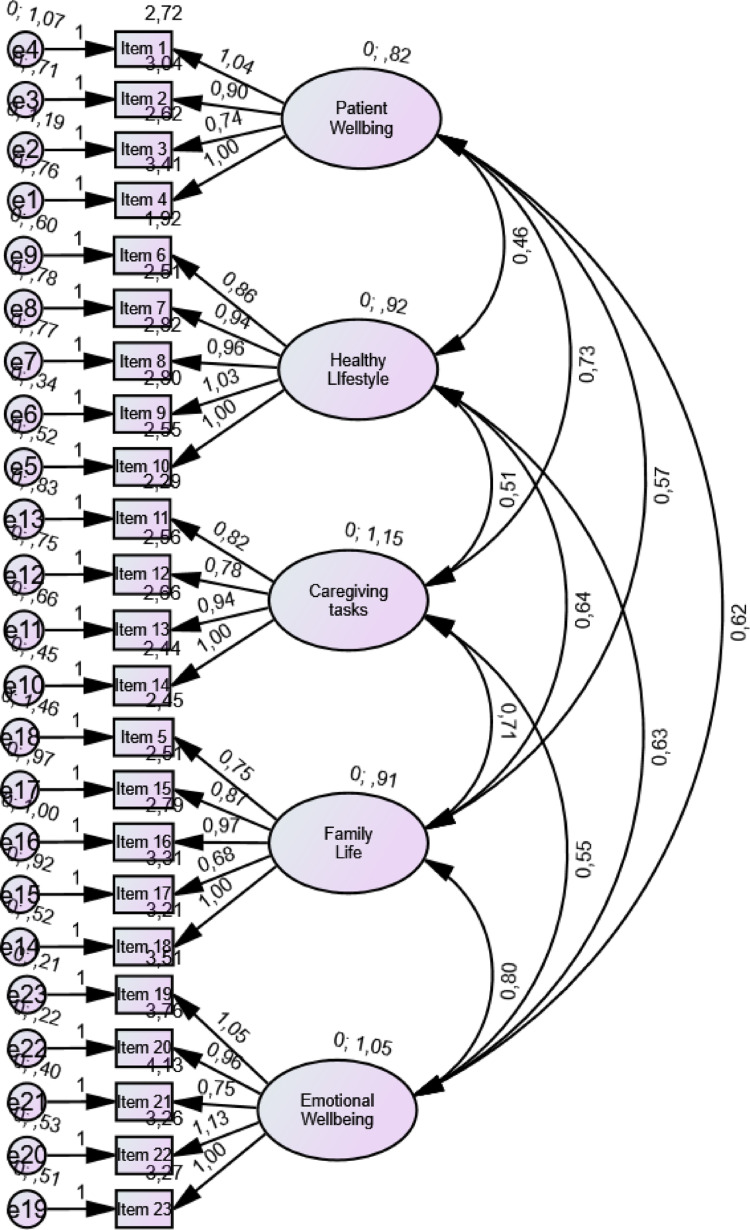
Confirmatory factor analysis using maximum-likelihood estimation of the hypothesized five-domain structure of the Swedish CSS-C among n = 145 Swedish family caregivers of persons diagnosed with cancer

Overall, the hypothesized five-domain model showed an overall acceptable, though not optimal, fit to the data, yet with indices of that it could be improved (Table 5, Fig. 1). The CMIN/DF, and SRMR indicated acceptable model fit. RMSEA (0.071) suggests a reasonable approximation of the population covariance structure, although the model does not meet the criteria for close fit. The CFI indicated acceptable relative model fit, whereas the GFI fell below the recommended threshold.

**Table 5:** Model fit measures with thresholds

Measure	Results	Thresholds
CMIN/DF	1.72	< 3 good*
GFI	0.819	> 0.95*
SRMR ≈	0.06	< 0.09*
RMSEA	0.071 (90% CI [0.059, 0.083])	< 0.05 good, 0.05–0.08 moderate, > 0.1 bad**
CFI	0.916	> 0.95 great, > 0.9 acceptable, and > 0.8 sometimes acceptable*

Evaluation of how well the hypothesized model fitted the data using maximum likelihood estimation. CMIN/DF = relative/normed chi-square statistics, GFI = goodness-of-fit statistics, RMSEA = the root mean square error of approximation, SRMR = standardized root mean square residual. CFI = comparative fit index

*Thresholds as recommended by Hu and Bentler (Hu and Bentler 1999). **Thresholds as recommended by Brown and Cudeck (Browne and Cudeck 1993)

### Convergent validity

A total of 14 items exceeded the threshold of 0.7, indicating that these items contribute meaningfully to their intended domain (Table 6). All but three of the remaining items exceeded 0.6. Item 3, “Changes in the patient’s memory or thinking”, Item 5 “Your relationship”, and Item 17 “Other family caregivers' well-being” represent the three items below 0.6, indicating limited convergent validity among these items. AVE values ranged from 0.42 to 0.73, with the *patient well-being* and *family life* being below threshold (0.5).

**Table 6:** Standardized factor loadings per item and average variance extracted per domain

Domain	Item	Standardised loading	AVE
Patient wellbeing	CSS_1	0.673	0.42
	CSS_2	0.693	
	CSS_3	0.524	
	CSS_4	0.720	
Healthy lifestyle	CSS_6	0.727	0.61
	CSS_7	0.717	
	CSS_8	0.724	
	CSS_9	0.861	
	CSS_10	0.801	
Caregiving tasks	CSS_11	0.692	0.58
	CSS_12	0.695	
	CSS_13	0.779	
	CSS_14	0.849	
Family life	CSS_5	0.509	0.42
	CSS_15	0.644	
	CSS_16	0.679	
	CSS_17	0.557	
	CSS_18	0.798	
Emotional wellbeing	CSS_19	0.920	0.73
	CSS_20	0.902	
	CSS_21	0.772	
	CSS_22	0.847	
	CSS_23	0.821	

### Discriminant validity

Inter-factor correlations between *patient well-being* and *caregiving tasks*, as well as between *family life* and *emotional well-being*, exceed the square root of AVE (Table 7). According to the Fornell and Larcker (1981) criterion, this suggests insufficient discriminant validity.

**Table 7:** Fornell–Larcker matrix of evaluations of discriminant validity of the domains of the Swedish CSS-C

	Patient wellbeing	Healthy lifestyle	Caregiving tasks	Family life	Emotional wellbeing
Patient Wellbeing	**0.648**	0.529	0.749	0.664	0.674
Healthy Lifestyle	0.529	**0.781**	0.499	0.693	0.645
Caregiving Tasks	0.749	0.499	**0.762**	0.694	0.498
Family Life	0.664	0.693	0.694	**0.648**	0.818
Emotional Wellbeing	0.674	0.645	0.498	0.818	**0.854**

The square root of average variance extracted is shown on the diagonal. Correlations are shown off-diagonal

### Reliability

Cronbach’s alpha for the total scale was 0.93, and across domains, it ranged from 0.76 to 0.93 (Table 3), indicating high internal consistency in the sample, yet with indices of redundancy. The *emotional well-being* domain had an alpha value of 0.93. Domain homogeneity values ranged from 0.39 to 0.72, all exceeding the predefined threshold and thereby indicating acceptable homogeneity.

## Discussion

We evaluated the validity and reliability of the Swedish CSS-C among 145 Swedish family caregivers of persons diagnosed with cancer. Overall, the Swedish CSS-C demonstrated satisfactory psychometric properties and criterion validity, despite limitations related to factorial distinctiveness and item redundancy.

As noted above, the Swedish CSS-C comprises 24 items, whereas the original versions of CSS-C comprised 33 (Shaffer et al. 2019) and 19 (Zaleta et al. 2023) items, respectively. Previous questionnaires and tools aimed at capturing family caregivers’ support needs both exceed and succeed 24 items; see, for instance, the Carer Support Need Assessment Tool (Alvariza et al. 2018) and the Supportive Care Needs Survey—Partners and Caregivers (Girgis et al. 2011), which comprise 14 and 45 items, respectively. Accordingly, there may be additional concerns that are relevant to family caregivers but that are not included in the Swedish CSS-C. For instance, the Supportive Care Needs Survey—Partners and Caregivers (Girgis et al. 2011) and the CSS-C 33-item version (Shaffer et al. 2019) include items addressing sexuality or spirituality. These were not included in the CSS-C 19-item version, nor in the Swedish version, but could be considered in future revisions.

In our study, the 24-item version demonstrated satisfactory psychometric properties overall. Both per item (Table 2) and for the items in their allocated domains (Tables 3 and 4). However, the evaluations of internal validity and internal consistency indicated redundancy among some items. Particularly within the *emotional well-being* domain and the anxiety and depression subscales. The Cronbach’s alpha for the total scale was 0.93 (Table 3), indicating strong internal consistency but also potential overlap among items. However, the scale is multidimensional, and the alpha value across domains ranged from 0.75 to 0.88, apart from the *emotional well-being* domain, which had an alpha of 0.93. In the development of screening tools and questionnaires, decisions regarding item inclusion inevitably involve balancing empirical evidence, psychometric performance, intended use, and practical feasibility while avoiding unnecessary redundancy (Streiner and Norman 2014). We suggest further evaluation of the included items, including exploration of item reduction in larger samples, using modern test theory and Rasch measurement theory to identify the optimal balance.

In the *emotional well-being* domain, all items exceeded the thresholds for acceptable item-total correlations (0.744–0.878) (Table 3). This domain comprises five items, four of which constitute the two anxiety and depression symptoms subscales: Item 19, “Feeling sad or depressed”; Item 20, “Feeling nervous or afraid”; Item 21, “Worrying about the future and what lies ahead”; and Item 22, “Feeling lonely or isolated”*.* An analysis of Cronbach’s Alpha If Item Deleted indicated that the internal consistency of the domain would increase if Items 19 and 20 were removed. At the same time, evaluations of the criterion validity demonstrated satisfactory correlations between the Swedish CSS‑C anxiety and depression subscales and the corresponding HADS subscales (0.720 and 0.757, respectively, Table 5), supporting the intended function of these two items. Notably, there were also strong cross-domain correlations. Although it could be argued as expected, given their empirically close interconnectedness (Kim and Carver 2019), this limits the criterion validity. Still, given the high inter-item correlations within the *emotional well‑being* domain, the contribution of individual items to the underlying construct may be questioned from a psychometric perspective (Streiner and Norman 2014; Prinsen et al. 2018). As mentioned, four of the domain’s items constitute two brief subscales designed to screen for symptoms of anxiety and depression. In contrast, the well-established HADS (17) consists of seven items per subscale. Despite the indicated redundancy of the Swedish CSS-Cs´ Items 19 and 20 within their allocated domain and of the domain itself, careful consideration is still needed before excluding any of the items. Therefore, no items were excluded in the present version. Nevertheless, the observed redundancy warrants further investigation using larger samples.

For construct validity, the model demonstrated an overall acceptable, though not optimal, fit, indicating that the specified structure provides a reasonable approximation of the observed data while leaving room for improvement. The CMIN/DF value (1.72) was below the recommended threshold of 3, suggesting an adequate balance between model fit and complexity. The SRMR (≈ 0.06) indicated good absolute fit, reflecting small residual discrepancies between the observed and reproduced correlations. The RMSEA value (0.071; 90% CI [0.059, 0.083]) fell within the range typically considered moderate or reasonable rather than close fit (Browne and Cudeck 1993). Although the lower bound approached the close-fit threshold, the upper bound slightly exceeded conventional cutoffs, suggesting modest approximation error rather than substantial misspecification. Given RMSEA’s sensitivity to sample size and degrees of freedom—particularly its tendency to overestimate misfit in smaller samples (Kenny et al. 2015)—it should be interpreted cautiously and alongside the other indices. The CFI (0.916) exceeded the threshold for acceptable fit, indicating a substantial improvement over the null model, whereas the GFI (0.819), known to be sample-size dependent, fell below recommended cutoffs. Taken together, and with consideration of the theoretically derived domains and the intended use of the Swedish CSS-C, the fit indices indicate an overall acceptable model fit, while also reflecting limitations related to sample size and approximation error. At the same time, domains comprising conceptually overlapping items, such as emotional well-being, may involve residual associations that are not fully accounted for by the latent factors. Future research using larger samples could therefore explore theoretically justified structural refinements, such as permitting correlated residuals between closely related items.

For convergent validity, *patient well-being and family life* did not fully meet the recommended AVE threshold of 0.50 (AVE = 0.42 for both constructs). This indicates that, on average, less than half of the variance in the indicators is explained by the latent constructs. However, most standardized factor loadings were moderate to high, with three items approaching the 0.50 threshold (Table 6), suggesting borderline but acceptable convergent validity when considered alongside theoretical relevance. For discriminant validity, the correlations between the *emotional well-being* and the *family life* domains and *patient well-being* and *caregiving tasks* exceeded the standard of acceptable threshold and the Fornell and Larcker (1981) criterion, suggesting insufficient discriminant validity. Empirically, the close connections can be explained. For instance, a close connection between affected family life and emotional well-being among family caregivers of persons with cancer is plausible (Nalbant et al. 2021). Likewise, the connection between patient well-being and caregiving tasks. Still, from a psychometric perspective, such high correlations raise questions regarding the degree of conceptual distinctiveness between these domains (Streiner and Norman 2014; Prinsen et al. 2018). As reported in the introduction, the *family life* domain was developed in the Swedish version through expert panels comprising family caregivers and healthcare professionals, hence, this overlap may have been introduced by adding this domain during the cultural adaptation. Still, given the overall success of the *family life* domain (Tables 2 and 3), supported by empirical evidence and its intended use, the five-domain structure of the Swedish version will be retained until it has undergone further evaluation in larger samples using modern test theory and Rasch measurement theory. In such evaluations, we suggest exploration of the relevance of retaining these domains as four distinct domains. The *healthy lifestyle* domain showed satisfactory convergent and discriminant validity (Tables 6 and 7), which is assuring given the intended purpose of the S-CSS-C. Overall, the evaluations (Table 5) indicate that domain scores should be interpreted as interconnected areas of need rather than strictly independent constructs. Accordingly, we find the instrument is most appropriately used as a brief screening tool, used to inform further assessment and dialogue, rather than to support definitive domain-specific decisions or as a comprehensive need assessment.

### Limitations

We used classical test theory to evaluate the validity and reliability of the Swedish CSS-C. While applying modern test theory approaches, such as Rasch measurement theory, could provide a more detailed understanding of item functioning and response-category performance, such analyses require larger sample sizes (Hobart and Cano 2009). Nevertheless, classical test theory provides well-established methods and criteria for evaluating validity and reliability. A limitation is the relatively small sample size (*n* = 145). Despite the COSMIN checklist suggesting a sample of at least 100 participants is sufficient to yield methodologically sound estimates (Gagnier et al. 2021) the CFA is more sensitive to small samples. Despite the measures of model fit reported in this study performing well with small samples (Hooper et al. 2008), RMSEA and GFI are still known to be sensitive.

We designed this study based on the literature on family caregivers’ experiences and needs. Literature highlights that despite the patient´s diagnosis and treatment may impact on family caregivers´ needs, there are similarities across diagnoses and settings (Baudry et al. 2019; Molassiotis and Wang 2022; Wang et al. 2018). These include, for instance, family caregivers tend to prioritize the ill person's needs over their own, and difficulties balancing family caregiving with overall family and everyday life. Thus, we did not collect data on patients’ cancer diagnoses, treatments, or prognoses since individual family caregiver factors were considered more relevant than cancer diagnosis and treatment (Krishnasamy et al. 2022), underscoring the need for individualized support. Still, we recognize that this data could have provided further information about the population among which the Swedish CSS-C was evaluated, which is essential in classical test theory (Streiner and Norman 2014). Importantly, within the sample, 84.8% are female and 66.9% university-educated**.** This composition may affect the representativeness of targeting and scaling findings across socioeconomic and educational gradients, which requires consideration and, preferably, further evaluation in more heterogeneous samples.

Finally, data were collected using an anonymous public survey, which allows multiple responses from a single person. No duplicate checks were performed, which is a limitation of this study. At the same time, this approach facilitated broader participation and enabled data collection without increasing the workload of the healthcare professionals in the participating cancer clinics. This trade‑off was considered acceptable given the exploratory and psychometric focus of the study. However, it must be recognized that this approach may disproportionately attract family caregivers who are more engaged or actively seeking support, potentially limiting the sample's representativeness.

## Conclusion

This study provides initial evidence supporting the validity and reliability of the Swedish CSS-C when used among family caregivers of persons diagnosed with cancer. Overall, the instrument demonstrated generally satisfactory psychometric properties, promising criterion validity, and an overall acceptable, though not optimal, factorial structure. The findings suggest that the Swedish CSS-C captures clinically meaningful and relevant areas of caregivers’ support needs. At the same time, the results indicate areas warranting further refinement. Evidence of item redundancy, particularly within the *emotional well-being domain,* and high correlations between several domains raise questions regarding the degree of conceptual distinctiveness among constructs. Accordingly, as of today, the Swedish CSS-C may be most appropriately used as a brief screening tool to guide further assessment and dialogue, rather than as a clinical decision tool or a comprehensive measure of caregiver needs. Future research with larger and more diverse samples is needed to further evaluate the scale’s structure and item functioning, including exploration of potential item reduction, alternative domain configurations, and theoretically justified structural refinements using modern test theory approaches. Such work will be important to optimize the balance between psychometric robustness, conceptual clarity, and practical feasibility in the continued development of the Swedish CSS-C.

## Data Availability

The datasets used during the current study are available from the corresponding author on reasonable request.
